# A Q fever cluster among workers at an abattoir in south-western Sydney, Australia, 2015

**DOI:** 10.5365/WPSAR.2016.7.2.012

**Published:** 2016-11-14

**Authors:** Heidi Lord, Stephanie Fletcher-Lartey, Guy Weerasinghe, Meena Chandra, Nilva Egana, Nicole Schembri, Stephen Conaty

**Affiliations:** a Public Health Unit, South Western Sydney Local Health District.; b Greater Sydney Local Land Services.

## Abstract

**Background:**

In September 2015, the Public Health Unit of the South Western Sydney Local Health District was notified of two possible Q fever cases. Case investigation identified that both cases were employed at an abattoir, and both cases advised that co-workers had experienced similar symptoms. Public Health Unit staff also recalled interviewing in late 2014 at least one other Q fever case who worked at the same abattoir. This prompted an outbreak investigation.

**Methods:**

The investigation incorporated active case finding, microbiological analysis, field investigation and a risk factor survey. Included cases were laboratory definitive or suspected cases occurring from October 2014 to October 2015, residing or working in south-western Sydney. A suspected case had clinically compatible illness, high-risk exposure and was epidemiologically linked to another confirmed case. A confirmed case included laboratory detection of *C. burnetti*.

**Results:**

Eight cases met the case definition with seven confirmed (including a deceased case) and one suspected. The eight cases were all males who had been employed at an abattoir in south-western Sydney during their incubation period; symptom onset dates ranged from November 2014 to September 2015. Field investigation identified multiple potential risk factors at the abattoir, and the majority (75%) of employees were not vaccinated against Q fever despite this high-risk setting.

**Conclusion:**

This cluster of Q fever in a single abattoir confirms the significance of this zoonotic disease as an occupational hazard among persons working in high-risk environments. Implementation of Q fever vaccination programmes should eliminate Q fever in high-risk occupational settings.

## Introduction

Q fever is a zoonotic disease caused by *C. burnetii*. (*1*-*7*) The main reservoirs for transmission of Q fever are cattle, sheep and goats. (*8*-*10*) Humans are predominately infected through inhalation of airborne dust or droplets containing *C. burnetii* bacterium. (*5*-*7*) The Q fever incubation period is 14 to 21 days. Q fever cases can present as either acute or chronic clinical manifestations; however, approximately 60% of Q fever infections are asymptomatic. (*5*-*7*) During the acute phase, symptoms are generally limited to a febrile illness with associated headaches, fatigue and chills. (*1*-*3*) Diagnosis of Q fever is predominantly through serological testing. (*1*, *3*)

In Australia, human infection with Q fever has been largely attributed to close contact with cattle, sheep and goats, particularly their reproductive organs and secretions. Those at greatest risk of Q fever are people employed at abattoirs, cattle farms and veterinarian clinics. (*1*-*7*) There have been 12 significant reported outbreaks of Q fever since 1959 with 9 of these associated with abattoirs, meatworks and cattle/goat/sheep farms. (*11*) In 2012–2014, a large outbreak of Q fever in Victoria was linked to a goat and sheep dairy farm with 18 confirmed cases over the period. (*12*) A safe and effective Q fever vaccine has been available in Australia since 1989. (*4*) It is recommended in the Australian Immunization Handbook (*13*) and mandated by SafeWork NSW (a New South Wales [NSW] government agency for work health and safety regulations) (*14*) for those employed in high-risk occupations.

In NSW, Q fever is a notifiable condition under the Public Health Act 2010 and notifiable to the local Public Health Unit (PHU). In September 2015, the South Western Sydney Local Health District (SWSLHD) PHU was notified of two possible Q fever cases. Both cases were interviewed and followed up according to NSW Health control guidelines for Q fever. (*15*) These interviews revealed that they had been employed at the same abattoir during their incubation period with no other likely risk exposures identified. These cases reported that co-workers had experienced similar symptoms. PHU staff recalled interviewing at least one other Q fever case in late 2014 who worked at the same abattoir. The interviews prompted further investigation to identify any additional possible or confirmed Q fever cases not notified to the PHU. This paper describes the approaches used in the Q fever cluster investigation and the findings that can inform Q fever surveillance and future investigations.

## Methods

The two Q fever cases notified to PHU were investigated. In addition, active case finding was conducted through (1) the line listing of abattoir employees, (2) routine case notifications, (3) local facsimile back system (facsimile sent to medical practices by PHU and sent back to PHU by the general practitioner (GP) with required information completed), (4) retrospective review of clinical pathology submissions from September to November 2015 together with field investigation around the abattoir, and (5) a risk factor survey. The additional cases identified through active case finding were investigated.

### Active case finding

1. The abattoir provided a list of all persons employed at the facility during the suspected exposure period. Further information including employees’ Q fever vaccination status (if not vaccinated, reason for not being immunized), occupation, duration of employment, and whether employees had a history of illness consistent with Q fever were recorded in the form of a line listing.
2. A retrospective review of Q fever cases notified to the PHU through electronic and paper-based reporting from the laboratories was conducted. The notifiable conditions database and all cases notified to PHU throughout the study period were reviewed. Review of symptom profile, possible risk exposures and laboratory methods were included.
3. General practitioners in the local government area surrounding the abattoir in south-western Sydney were contacted and asked to review any possible Q fever cases who presented to their practices. This process was implemented through a local facsimile back system. Written permission to contact these cases for investigation was provided by the GP.
4. A retrospective review of Q fever clinical pathology submissions during the study period for a resident of SWSLHD (identified using residential postcodes) was performed with the assistance of the State reference laboratory, NSW Pathology West, previously Institute for Clinical Pathology and Medical Research (ICPMR).

Additional cases were identified via cross-referencing of the above line listing with cases already notified to the PHU.

### Case definition

We referenced the NSW Control Guidelines for Q fever to define the cases in this investigation: (*15*)

1. A suspected case was defined as any person who had clinical evidence of Q fever (fever, headaches, fatigue, chills), a high-risk exposure to *C. burnetii* and was epidemiologically linked with other suspected or confirmed cases in the cluster.
2. A confirmed case was defined as any person who had:
  - laboratory-definitive evidence:
    - detection of *C. burnetii* by nucleic acid testing, or
    - seroconversion or significant increase in antibody level to Phase II antigen of *C. burnetii* in paired sera tested in parallel in the absence of recent Q fever vaccination, or
    - detection of *C. burnetii* by culture; or
  - laboratory-suggestive evidence (i.e. detection of specific IgM in the absence of recent Q fever vaccination) and clinical evidence of Q fever disease.

### Laboratory methods

Commercial enzyme immune assays were used for initial serological testing by detecting Q fever IgM and IgG antibodies. Results of NSW Pathology West laboratory testing were requested to reach a definitive diagnosis. NSW Pathology West tested acute and convalescent specimens using immunofluorescent antibody testing and complement fixation testing for both Phase 1 and 2 antigens.

### Risk factor survey

A modified risk assessment section of the standard Q fever investigation question package (*15*) was developed, which included additional questions to capture potential risk factors for Q fever. Cases were asked about symptom profile, occupational risks, vaccination and exposure to animals outside of their occupational setting.

### Field investigation

An inspection of the affected abattoir conducted on 13 October 2015 involved review of abattoir documentation encompassing standard operating procedures for new staff inductions, work health and safety regulations and vaccination for Q fever; gathering information on species slaughtered and wholesalers who provide them to the abattoir; inspection of the kill floor, holding yards and layout and design of the abattoir; and review of cleaning practices. Staff knowledge of Q fever was also assessed by asking questions about transmission, vaccination, symptoms and their understanding of abattoir management reporting requirements for Q fever.

## Results

In total, we identified eight cases of Q fever (seven confirmed and one suspected cases) with onset dates ranging from 24 November 2014 to 9 September 2015 (**Table 1**). All cases were males employed at the implicated abattoir during their incubation period. Most cases had fever (7/8), followed by lethargy and malaise (6/8), headache (5/8), chills or rigors (5/8) and nausea and vomiting (5/8) (**Table 2**). Case 7 was seen by a GP and was deceased on arrival to a hospital three weeks after onset of symptoms. A coronial inquest into his death indicated that Q fever was a significant condition contributing to his death but not the condition causing his death.

**Table 1:** Summary of confirmed and suspected cases in the Q fever cluster, south-western Sydney, Australia, 2015

Case No.	Age, Sex	Onset date	Notification date	Laboratory evidence	Investigation classification	Method used to identify case
1	17, M	24/11/2014	10/12/2014	Definitive seroconversion	Confirmed	RR*
2	28, M	27/11/2014	09/01/2015	Definitive – nucleic acid testing	Confirmed	RR
3	28, M	28/11/2014	08/09/2015	Definitive seroconversion	Confirmed	I
4	22, M	11/01/2015	13/10/2015	Suspected case (no convalescent sample available)	Suspected case	RR
5	27, M	27/07/2015	30/11/2015	Definitive seroconversion	Confirmed	RR
6	17, M	31/08/2015	21/10/2015	Definitive seroconversion	Confirmed	A
7	60, M	07/09/2015 (deceased 30/9/2015)	18/09/2015	Definitive seroconversion	Confirmed	I
8	45, M	07/09/2015	21/10/2015	Definitive seroconversion	Confirmed	A

Note: A: Abattoir line listing, I: Initial case/s that prompted the investigation, RR: Retrospective Review of Laboratory Reporting.

* Public Health Unit staff recalled being notified of this case after being notified of cases 3 and 7.

**Table 2:** Symptoms reported by confirmed and suspected cases in the Q fever cluster, south-western Sydney, Australia, 2015

Symptom/Abnormal investigation findings	Number of cases	%
Abnormal liver function tests Endocarditis Fever Headache Chills or rigors Lethargy and malaise Abdominal pain Nausea/Vomiting Arthalgia/Myalgia	4 0 7 5 5 6 1 5 4	50 0 87.5 62.5 62.5 75 12.5 62.5 50

Six cases were identified after active case finding; four cases through retrospective review of laboratory reporting to PHU and two through the abattoir line listing. Furthermore, one potential case was identified through GP facsimile back. However, this case did not meet the confirmed or suspected case definition for Q fever and was excluded.

Risk factor surveys were conducted between October 2015 and November 2015, which revealed that only 25% (2/8) of cases had previously received a Q fever vaccination (**Table 3**). All eight cases had high-risk exposures during their current employment: handling the carcasses/slaughtering of pregnant animals, contact with animals giving or having given birth recently, and handling of animal fetuses and waste containers used for collection and disposal of birthing products. None of the cases identified other potential risk factors outside their occupational setting. Numerous attempts to interview or have asymptomatic staff complete the risk factor survey were unsuccessful.

**Table 3:** Summary of findings from the risk factor survey among confirmed and suspected cases in the Q fever cluster, south-western Sydney, Australia, 2015

Assessment criteria	Number of cases
1. Current occupation at an abattoir	8/8
2. Experienced Q fever symptoms in past 12 months (combination of the symptoms including fever, severe headaches, muscle aches, extreme fatigue, joint pain, sweating and chills)	8/8
3. Received Q fever vaccine in the past	2/8
4. Tested positive for Q fever – blood test only	8/8
5. Doctor has advised ongoing check-ups/scans or blood tests	4/8
6. Worked in a high-risk occupation in the month before onset of symptoms (Yes = abattoir)	8/8
7. GP or hospital doctor ever requested an echocardiogram or heart scan due to symptoms	3/8
8. Still have problems/symptoms related to Q fever	5/7^#^
9. Type of work done in abattoir	
a. Slaughtering	8/8
b. Boning	2/8
c. Packing	2/8
d. Inspecting meat	1/8
10. Types of animals* in contact with as part of abattoir work	
a. Cattle	8/8
b. Sheep	8/8
c. Goats	8/8
d. Pigs	8/8
11. Contact with fluids from pregnant animals or animals giving birth	
a. Animals giving birth	4/8
b. Handled carcass/slaughtering of pregnant animal	6/8
c. Handling of animal fetus or slops bucket	3/8
12. Family member living in the same house as case working in an abattoir	3/8
13. Time lapse before seeing a doctor after first symptoms developed	
a. Immediately to within two weeks	6/8
b. Between two weeks and six weeks	2/8

* Only these species are processed at this abattoir.

^#^ Does not include a response from the deceased.

Field investigation at the abattoir identified that there were 33 staff currently employed at the abattoir – 23 were employed to slaughter animals; the other 10 staff had roles in management, maintenance and stock handling. Management advised that there was a high turnover of staff. High turnover of staff and the ongoing pressure of needing to start employees immediately was certainly a concern for the abattoir management and could have potentially contributed to the problem of occupational vaccination for Q fever. Liaison with abattoir management was challenging, and low compliance with appropriate work health and safety obligations was evident. The field investigation revealed that management and staff were lacking in knowledge and awareness of Q fever infection. Abattoir management were not compliant in reporting to SafeWork NSW.

Possible high-risk exposures included animals aborting/giving birth in the holding yards and at the evisceration point where a fetus (if identified) would be pulled out and dumped into a slops chute; however, it was difficult to ascertain where these infectious materials were stored or disposed. All staff on the kill floor would have potentially been exposed to the aerosolization of the birthing products. Additionally, staff were observed smoking during their break times, indicating a possible hand-to-mouth exposure if strict personal protective equipment (PPE) and hand hygiene practices were neglected.

In keeping with NSW Health Q fever control guidelines, the SWSLHD PHU made a formal notification of the Q fever cluster to SafeWork NSW, the enforcing body for Work Health and Safety Regulations. Further follow-up with SafeWork NSW confirmed that the abattoir was issued a strict warning and a recommendation to implement a vaccination programme for existing and future staff.

## Discussion

This was a significant cluster of Q fever in a high-risk setting. This outbreak in south-western Sydney compares with several previous outbreaks in both size and case finding but particularly the abattoir outbreak in South Australia in 2007 with five confirmed cases and one possible fatality. (*16*) This investigation has confirmed the significance of this zoonotic disease as an occupational hazard for people working in high-risk settings and underscores the need for accurate diagnosis and timely reporting. It has also highlighted the challenges of a public health investigation in an area where the legislative enforcement authority lies with other agencies and demonstrates the need for improved interagency communication.

The application of active case finding strategies created the opportunity to identify potential cases in the community and within the vicinity of the abattoir – especially given that the field investigation identified various vulnerable groups (a school and residential properties) within close proximity to the abattoir. This was important since Q fever infection, which can be prevented by controlling the disease at its source, can be asymptomatic in approximately 60% of cases. An outbreak in the Netherlands in 2007–2010 was thought to be associated with intensive dairy goat farming that reported an increased number of abortions in the years before the first human cases. (*17*, *18*) Cases were found to be residing within close proximity to the farms (5 km radius) that were thought to be the primary source of infection precipitated by the dry weather aerosolizing *C. burnetti*. (*17*, *19*, *20*) This demonstrates the necessity for surveillance and active case finding in the area surrounding an abattoir. It is important to note that only looking for symptomatic cases may grossly underestimate the number of exposures associated with an outbreak as was demonstrated by the Dutch experience.

Although the PHU was notified of the first case in December 2014, limitations in the surveillance process may have inadvertently prevented the detection of other cases in a more timely fashion. Timely notification of positive results from laboratories and an alert system in the notifiable conditions database may have notified PHU staff to the cluster earlier.

Issues in this study arose with the absence of clear guidelines to notify, collaborate with or provide recommendations for interagency communication. This investigation also revealed the alarming lack of knowledge among abattoir management and staff about the risk of Q fever. Equally disconcerting was the absence of a prescreening and vaccination programme. The abattoir has a responsibility to ensure all staff, before commencement of employment, attend a health-care provider to carry out the prescreening process that requires checking immunization records for evidence of Q fever vaccination or screening for previous exposure to Q fever through skin and blood testing to rule out contraindications for vaccination. Such programmes are imperative not only for detecting possible exposure/cases, but also for identifying persons for which the Q fever vaccine is contraindicated because of previous infection or vaccination. (*13*) Poor recordkeeping at the abattoir made it difficult to identify previous staff and the roles they occupied during their period of employment at the abattoir. This issue also made establishing the immunization status of current or previous employees at the abattoir extremely challenging. A lack of cooperation from asymptomatic staff to complete surveys or be interviewed also limited the information that could be collected.

The abattoir has a duty of care and legal obligation to their employees given the high-risk occupational setting. Other outbreaks have demonstrated that the optimal time period for Q fever vaccination is two weeks before possible occupational exposure. (*14*, *21*) SafeWork NSW guidelines indicate an employer must implement safe work practices to minimize risk and notify SafeWork NSW if one of their employees has Q fever. (*14*) This case investigation concluded that despite abattoir management being aware of several employees with Q fever symptoms, not even the death of an employee linked to Q fever prompted appropriate notification. Although a warning and compliance order was issued to the abattoir, this action is not comparable to restrictions placed on abattoirs in previous outbreaks and may not mitigate any ongoing risk to employees. In previous abattoir outbreaks, restrictions had been placed on the abattoir operation (including access restriction to those who could not show evidence of vaccination, erection of biosecurity signage on all access roads to the abattoir/farm, introduction of vehicle wash stations and foot baths, changes to work health and safety policy at the facility and introduction of uniforms with laundering onsite with a longer-term plan to develop showering facilities onsite) along with recommendations for a mandatory vaccination programme for all staff in these high-risk settings. (*14*, *22*) Increased monitoring by agencies responsible for work health and safety may be necessary to ensure prescreening and vaccination programmes and other necessary restrictions and policies are implemented for employees in high-risk occupations. An area of further research would be to assess the level of noncompliance with work health and safety legislation in abattoirs across NSW.

### Limitations

This study had several limitations. The risk assessment survey was conducted 12 months after the initial onset of symptoms in some of the cases with the possibility of recall bias due to the time lapsed. Lack of resources and time constraints prevented the expansion of the investigation to neighbouring residences and schools, which might have resulted in an underestimation of the scope of the outbreak. However, the retrospective review of the pathology results was used as a proxy for this. While this investigation demonstrated great collaboration between human and animal health experts, reliance on one agency for the field investigation may have limited the information obtained from the abattoir. Development of a checklist for future field investigations could be explored to alleviate this limitation. The study is also limited due to the inability to access information on other abattoir workers who were not diagnosed or tested to provide a comparison. This study must therefore be interpreted in the context of a case series.

## Conclusion

This investigation revealed that Q fever is a significant zoonotic disease, especially among abattoir workers, and underscores the need for accurate diagnosis and timely reporting. In high-risk settings, prescreening and vaccination programmes are imperative prevention strategies, which require close collaboration between public health and agencies responsible for work health and safety to ensure maximum compliance.

This investigation highlights the need for multiagency review of the management of Q fever in these high-risk settings, especially in regards to notifications to PHUs and adherence to work health and safety regulations.
